# Factors influencing sexual harassment behavior in sports environment: Evidence from Pakistan

**DOI:** 10.3389/fpsyg.2022.837078

**Published:** 2022-11-17

**Authors:** Nabila Sarwar

**Affiliations:** Sports Sociology, School of Physical Education and Sports Sciences, South China Normal University, Guangzhou, China

**Keywords:** organizational climate, hostile sexism, low self-esteem, female athletes, sexual harassment

## Abstract

Sexual harassment (SH) in sporting environments has developed as an important field of research. However, a more comprehensive understanding of factors affecting SH behavior is needed. We hypothesized how these effects appear and the scenarios in which they exist. Based on the perspectives of SH, this study aims to reveal the athletes’ perceptions of SH and organizational climate (OC) impacts on SH, as well as the mediating mechanisms of low self-esteem (LSE) and the moderating role of hostile sexism (HS). Data collected from 422 female athletes in Pakistan, using random sampling and using SEM techniques analyze that these factors are responsible of increased likelihood of female athlete SH. Our results suggest that athletes’ perceptions of OC positively predict SH, and OC is positively related to LSE. In addition, LSE partially mediates the positive effects of athletes’ perceptions of OC on SH, where HS strengthens the perceived relationship between OC and LSE, which stimulates the SH of athletes. The overall model predictive ability was carried out by moderated mediation model. This study had deep implications for the literature. It clarified the ways that are crucial for predicting OC. Whether there are written laws on the subject, organizations are reluctant to acknowledge them, which entails developing methods of quantifying SH and creating data accessible to the public. A future study is recommended to evaluate the abuse of power concerning benevolent sexism to identify athlete perception of SH.

## Introduction

Every culture defines sexual harassment (SH) differently, related to the level of social acceptance and perception of the act (Espelage et al., 2016). As sport is a work field, the definition of sport is also derived from the workplace definition. However, we established boundaries under a particular term of sexual experiences questionnaire (SEQ), which is mostly used as an indicator of SH to date (Lonsway et al., 2013), and investigated three aspects of SH (Barreiro, 2020;Bartolo, 2021). Millions of women worldwide endure SH or must deal with its effects routinely (Watts and Zimmerman, 2002; UN Women, 2008). Women’s sports engagement is still regarded as inappropriate in many nations, particularly in Pakistan (Gul et al., 2019). Although sports can help to alleviate gender inequality and violence against women (Tariqa and Ishit Kochhar, 2021), SH can be seen in all sports at all levels, including professional sports (Fasting and Sand, 2015). In a combination of elements, organizational context (OC) is the foremost predictor of SH (DeSouza and Cerqueira, 2009), where social climate, abuse of power, poor law enforcement, male dominance, gender ratios (Engelberg and Moston, 2016b), patriarchal leadership, current tolerance systems and fear of retaliation cooperate to create an environment in which SH against athletes can evolve and continue (Vaux, 1993; Kirby et al., 2000; Fasting et al., 2014; Engelberg and Moston, 2016a).

To the best of our knowledge, there has been much data on SH in the workplace, but specifically in sports three studies found in Pakistan dealt with SH, including SH, coach molestation conduct and women’s role in sports. These three studies were prepared by Jahangir and Manzoor (2018), Gul et al. (2019), Bhatti et al. (2020), and Din et al. (2020), respectively. To date, no research has examined the sociocultural factors influencing sports females’ perception of SH from SH theories. This study will address this absence in the literature by providing insight into female athletes’ perception of SH in Pakistan, relying on accepted theories of SH, using SEM analysis (Kapila, 2017), examining the implicit effect of OC on SH, and incorporating the explicit psychological mechanisms of LSE among athletes and the moderating role of hostile sexism (HS), because HS paves a way to SH for both men and women and strengthens the relationship between LSE and OC in predicting SH. A relatively under-explored research area, we hope, is to broaden our knowledge of the subject. A significant relationship between the important drivers that influence the perception of female athletes of SH redounds to society’s benefit, because society is male-dominated and involves intoxication of power. This means that there is a dire need to change their mindsets and stereotypes about females and socialize their generation in a way where females get equal rights as their male counterparts, strengthening the bond of relationship among family athletes and coaches to boost the well-being and confidence of athletes automatically. When personal safety is violated, whether its men or women, they should use legal tools to protect themselves. Athletes and their parents should be informed of the risks of abuse and the means of legal action available to them, and punishments employ a lesson for others. Sports organizations’ staff should be provided with specific training and ensure that criminal and disciplinary provisions are applied. By devising such an operational methodology by looking into the past background of the harassment incidence, there might be a drastic fall in SH of females in sports. A drastic fall in SH may also happen when sports committees properly enforce all these implications. For the researcher, future research investigation will uncover a critical area in the SH that many researchers could not explore yet. Thus, an exploratory study on the sociocultural factors affecting SH may be arrived at. In the following section, the theoretical framework and review of the study are presented.

## Theoretical background and hypotheses

### Sexual harassment theories

The concept of SH differs from society to society, depending on both the victim and the perpetrator (Fasting and Sand, 2015). Previous scholars looked at SH using various approaches (Sarwar and Nauman, 2011). To investigate the factors that affect female athletes’ perception of SH in Pakistan, this study analyzes different theories of SH; the power relationship between the harasser and the harassed appears to be an appropriate perspective to employ, given the subject matter in particular, where the overall power structure within an organization is founded on masculinity (Samuels, 2003). Historically, cultures and societal norms have granted males the power to exercise dominance over women, exploiting their physical, economic, and political superiority (Tangri et al., 1982). Feminists relate SH to the sexist and patriarchal philosophy of male supremacy present in society (DeSouza and Fansler, 2003; Beech et al., 2006). This gender hierarchy is maintained through dominant normative masculinity and sexual characteristics (Cleveland et al., 2000; Burn, 2019). Organizational theory (Tangri et al., 1982) postulates that the prevalence of SH depends on systems of control and the exercise of power (Cleveland and Kerst, 1993). Feminism explains that organizations inevitably reflect societal gender hierarchies, which entails SH between coworkers or between managers and subordinates. As an extension of the organizational theories of SH, sex-role spillover (Gutek and Morasch, 1982) suggests that men express intense sexual appetites and play gender roles at work. They may stereotype women as a commodity instead of valuing them as coworkers. In male-dominated institutions, spillover effects explain gender ratios (Fitzgerald et al., 1999), where numerically fewer females are considered to deviate from established roles.

#### Research question

To investigate the factors affecting the SH behavior of female athletes in sports in Pakistan, through the mediating role of low self-esteem (LSE) and the moderating role of hostile sexism.

#### Hypothesis of the study

H1: Respondents’ attitudes toward OC are positively related to their perceptions of SH.

H2: LSE mediates the relationship between perceived OC and SH.

H3: HS moderates the relationship between perceived OC and LSE.

### Organizational climate and sexual harassment in sports

The sociology of sport existed for many years before any study of SH. Violence in sports became a central topic in the early 1990s. The field of sports is conceptualized as a male-dominated culture, which facilitates various forms of discrimination against female athletes, including SH perpetrated by coaches and male athletes (Dworkin and Messner, 2002). Scholars have reported that women are frequently subject to SH (Huerta et al., 2006). As the industry is deeply rooted in hyper-masculine tendencies (Sullivan, 2021), it is largely men who perpetrate SH (Parent et al., 2016; Ford and Ivancic, 2020; Sutton et al., 2021) and negatively influence females (Omorogiuwa, 2018). An organizational climate (OC) is an expression of individuals’ common views and mechanisms that explains how certain activities are geared toward achieving SH goals (Fasting et al., 2010). They provide norms of conduct and identify how individuals should behave in the organization, including environmental beliefs, principles, and assumptions (Ostroff et al., 2012). Several studies have independently analyzed OC as an indicator for SH, while others have shown that OC and job gender ratio are significant antecedents for SH (Willness et al., 2007). Many authors have found that an environment where SH serves male bonding exhibits organizational tolerance, male-dominant cultures, and sexually objectifying environments (Holland et al., 2016; Burn, 2019). In the world of sports education, the coach’s authority is rarely questioned by either parents or athletes (Huerta et al., 2006; Jahangir and Manzoor, 2018). Some have considered that SH can only come from authority figures, but another study by Sibley Butler and Schmidtke (2010) showed that gender-based behaviors are used in organizations to threaten women in all positions. In this context, the organizational power structure is often investigated (Cleveland and Kerst, 1993). According to Roehling and Huang (2018), in many sports organizations, it is a common belief that women are not suitable for coaching or leadership roles, and men are traditionally been viewed as natural for leadership positions (Walker and Sartore-Baldwin, 2013). This influence is generally more pronounced where sex-role behaviors match gender ratios in the organization in the workgroup (O’Leary-Kelly et al., 2000). Men tend to have key positions at higher levels of organizational governance and to have better sports than women (Taylor and Hardin, 2017). The perceived danger to victims of abuse, lack of sanctions against abusers, and the belief that allegations are not taken seriously have been given as reasons for SH (Jahangir and Manzoor, 2018). Following the literature, the Pakistani athlete’s perceptions of organizations are considered a leading cause of SH, because injustice and false accusations caused a cricketer to commit suicide in 2014. In this line of thinking, the study hypothesizes that female athletes’ perception of an OC would be significant for SH.

**H1: Respondents’ attitudes toward OC are positively related to their perceptions of SH.**

### Low self-esteem and sexual harassment in sports

Women are widely regarded as inferior to males in all facets of life, and sexism in sports has always been common. Characteristics potentially affect the maintenance of athletes’ self-esteem, and we grouped those individual factors into LSE, which may predict or influence SH. Risk factors of SH include the athlete, coach, and sports factors where athlete’s factors further break into a poor relationship with parents, LSE, and close relation to coach (Marks et al., 2011). The institutional climate of SH is profoundly rooted in OC and underpinned by the mediating role of LSE (Dworkin and Messner, 2002). Szymanski et al. (2011) explained that individual characteristics function as mediators of male misconceptions, such as aggressive masculinity, passionate sexual feeling, and alcohol use in dating and intimate circumstances. Females often do not dare to report to organizations due to intimate questions, family concerns, and the need for professional prestige (Ali and Kramar, 2015), all of which further damage self-confidence. Pakistan cricketers Shahdab and Yasir alleged SH cases but no action was taken (Morning Express, 2022). A dearth of knowledge of SH among athletes, inadequate physical evidence, failure of coach supervision, parenting stress, lack of physical evidence, lack of coach monitoring, parental rejection (Hill and Silva, 2005), and causes LSE. Sexual abuse by coaches is extremely toxic and distressingly common (Nelson, 2000). A common girl in India was often forced to conceal outrageous instances of abuse and SH, which caused a massive change in her strong sense of LSE (Ghosh, 2013). The suicide of cricketer Haleema Rafiq is another sad case of not taking accusations seriously (Naseer et al., 2016). Various negative life experiences, unworthy comments such as continuous criticism, and physical and verbal abuse can decrease self-esteem (Bowker, 2006). Fasting et al. (2002) reported that when the harasser was even at the same venue or sporting event, it became hard for the athletes to focus. These feelings allowed the harassers to gain strength in their relationship with SH (Smith, 2016). Leaper and Brown (2008) concluded that persistent SH adversely influences a girl’s self-esteem, body appearance, fitness, accomplishment, and confidence. Athletes tend to accept SH to balance the potential benefits of performance success (Huerta et al., 2006). When individuals with a propensity for SH are placed in social situations that permit or encourage this behavior, harassers are apt to indulge in SH, as they consider it acceptable due to their environment (Willness et al., 2007). As to what has been discussed above, we would suggest that the degree to which SH is widespread in the research areas is an individual aspect that plays a mediating role in the OC and SH.

**H2: LSE mediates the relationship between perceived OC and SH.**

### Hostile sexism and sexual harassment in sports

The unwritten rules followed by our society and culture do not enable female participation in sports due to the widespread interpretations of the norms of religion and society (Ashraf, 2019). Cultural variations produce different beliefs around SH in different countries and play a vital role in creating people’s attitudes and aspirations (Berdahl et al., 2015). HS is antagonism to women who transgress conventional expectations of gender. Those who threaten the traditional hierarchies of power are subjected to SH (Gruber and Fineran, 2016; Russell and Oswald, 2016). The complexities of gender relations and the notions of masculinity and femininity may result in gender divisions of various kinds; for instance, in some contexts, men may seek to show their masculinity by sexually harassing a woman (Burn, 2019). SH tends to justify patriarchal dominance, traditional gender norms, and coercive conduct by men toward women, treating them as sexual artifacts (Galdi et al., 2014) and retaining organizational roots. Eroding and antiquated norms regarding what males and females should do and should not do help members of both sexes understand their full potential (Ali and Kramar, 2015). A structure of gender discrimination can damage both women and men by promoting assumptions that hinder their capacity as individuals to fulfill their maximum potential (Berdahl et al., 2015). SH remains present in Pakistan in all sports, but social practices discourage women from reporting it because perpetrators of SH have never been convicted in the country (Jahangir and Manzoor, 2018). Historically, stereotyping societies and societal expectations have socialized men into considering themselves stereotypes of sexual affirmation, leadership, and persistence, while women are socialized as passive and submissive gatekeepers. Cultural perceptions of gender norms also appear in the workforce, culminating in the sexual objectification of female workers by their male coworkers and supervisors (DeSouza and Cerqueira, 2009). This behavior also dominates in sports organizations, which leads women to develop psychological stress and a low sense of self-worth (Samuels, 2003). The lower status of women in mainstream culture is reflected in the values and principles of sports institutions; thus, male dominance appears to be in common practice. Sport is one of the most widely recognized domains of male leadership (Taylor and Hardin, 2017). Feminists understand that certain characteristics that thrive in a male-dominated culture increase the probability of assault for everyone. Concerning the literature, levels of athletes’ perception of SH differ depending on HS (Russel and ve Trigg, 2004; Sandys, 2008). In this study, we tested HS to communicate between the OC and LSE concerning their impact on SH in sports settings.

**H3: HS moderates the relationship between perceived OC and LSE.**

## Materials and methods

### Study population and inclusion criteria

This study used a survey research design to achieve its research objectives. Twelve women’s football clubs were focused. The study population consists of Football Association affiliated with Pakistan Football Federation who were believed to be potential respondents.

### Sampling procedure and sample size

Random sampling techniques were used to select participants, according to the confidence level of 95% and confidence interval (significance level) of 5% (Munir et al., 2020). There is also an alternative of G-power analysis where *post hoc* test shows the medium effect size with 0.9 power and 0.05 CI. Concerning the method of approaching the participants (sports club athletes), instructors had given a link that took participants to the survey monkey online questionnaire. It has many advantages such as saving time, clarifying doubts of the respondents, the opportunity to motivate the respondents to offer true answers, and bearing fewer expenses (Sekaran and Bougie, 2016). Data collection was held during the lockdown period, and some areas were out of Internet access. Due to the low Internet availability in different regions of Pakistan, it was not easy to approach all girls through an online questionnaire. Thus, a research facilitator was recruited for the purpose of data collection. To reach the target population and to get the valuable data, authors and facilitators contacted different athletes and coaches, and printed questionnaires were sent to the women athletes. Data were gathered during the lockdown time in May and June 2020 from athletes aged between 18 and 35 years.

### Ethical considerations

This study was conducted with the recommendation of a human ethics committee of South China Normal University, China, SCNU-SPT-2021-125. Since the issue was susceptible, the President of the Football Association was also requested an ethically concerned written consent letter.

### Construction of variables

All the variables included in this study were measured using already-developed scales. The Sexual Harassment Scale of Fitzgerald et al. (1995) having 13 items was used to measure the players’ perceptions of SH, for example, (1) “female players encounter offensive and sexist remarks and body gestures” and (2) “repeatedly told awful sexual stories or jokes, overlook offensive sexual comments about their dress, body, or behavior.” The Organizational Tolerance of Ford and Ivancic (2020) with 9-item scale included statements like (1) “My organization doesn’t want employees to come forward about sexual harassment.” Similarly, the Ambivalence Sexism Inventory (ASI) of Flske and Glick (1995) for Hostile Sexism with 11-item sub-scale was taken as a measure having statements like (1) “Feminists are making entirely reasonable demands of men.” Finally, the Texas Social Behavior Inventory (TSBI) of Helmreich and Stapp (1974) for LSE with 13 items was established including statements like (1) “Females less confidently approach and deal with perpetrator behavior.” In this way, this study assessed the athletes’ perceptions through a well-organized questionnaire in English language using an online link. However, it was essential to pretest the questionnaire before using it for data collection, and a pilot study was carried out with two clubs. The main aim of the pretest is to refine the questionnaire and enable the researcher to obtain an assessment of the validity and reliability of the questions (Thornhill et al., 2009).

Initially, two researchers from the Harran University, Turkey, and the International Islamic University of Pakistan, who are assistant professors and have knowledge of the background, are being requested for clarity, ease of reading, and appropriateness of the questionnaire before survey distribution (Thayer-Hart et al., 2010).

They were asked to rate the degree to which they agreed or disagreed using a five-point Likert scale (Graziano and Raulin, 2013).

### Data cleaning

Before the analysis, data cleaning was assessed, and normality, linearity, homoscedasticity, and multicollinearity assumptions were met. After the detection and exclusion of univariate and multivariate outliers by checking frequencies and distributions, a sample size of 422 was considered appropriate. It also helps to ensure that the data are valid, reliable, and representative. The link has been forwarded to 480 females in total. However, 435 completely filled-in questionnaires were received.

### Analysis of data

For data analysis, this study used SPSS and AMOS software. Correlation analysis, confirmatory factor analysis (CFA) (due to some changes in the original questionnaire according to the scenarios of this study, we need to regenerate the reliability and validity of the questionnaire), mediation analysis, and moderation analysis were performed.

## Results and interpretation

### Descriptive statistics

The arithmetic mean (AM) and standard deviation of each latent variable were calculated. The AM results showed that average responses were between 2.5 and 3.4. Therefore, the responses were equally distributed from “1” (strongly disagree) to “5” (strongly agree). Participants who scored higher showed higher levels of perception about study variables. The SD indicated the unit change in average responses.

### Measurement model (exploratory factor analysis)

To validate the factors that are being used in this study, exploratory factor analysis (EFA) was conducted. Bartlett’s test of sphericity was [χ^2^ (741) = 10,195, *p* < 0.05], the KMO sampling adequacy threshold value was 0.95 > 0.70, and the total variance explained by all four factors was 56% (Hair et al., 2010), suggesting that the items were factorable. This indicates that the factor ability of R assumption was good, and the eigenvalues of factors were > 1. The authors deleted items (LSE = 6.9.10.12.13, SH 13, and HS 3) with factor loading < 0.50 and correlated with other items (Hair et al., 2006). Excluding these problematic items, EFA confirmed the four factors, i.e., OC, LSE, HS, and SH, based on the abovementioned criteria.

### Common method bias

Examining the common method bias of the instrument is necessary because the constructs are extracted from different research studies, and data were collected in an online survey that may introduce systematic response bias. So, the threshold criteria for no common method bias were proved by this. Harman’s one factor 31.07% (< 50%) (Jiang et al., 2018) method is used to test the common method bias (Podsakoff et al., 2003) (see Table 1B).

### Results of measurement model (confirmatory factor analysis)

**FIGURE 1:**
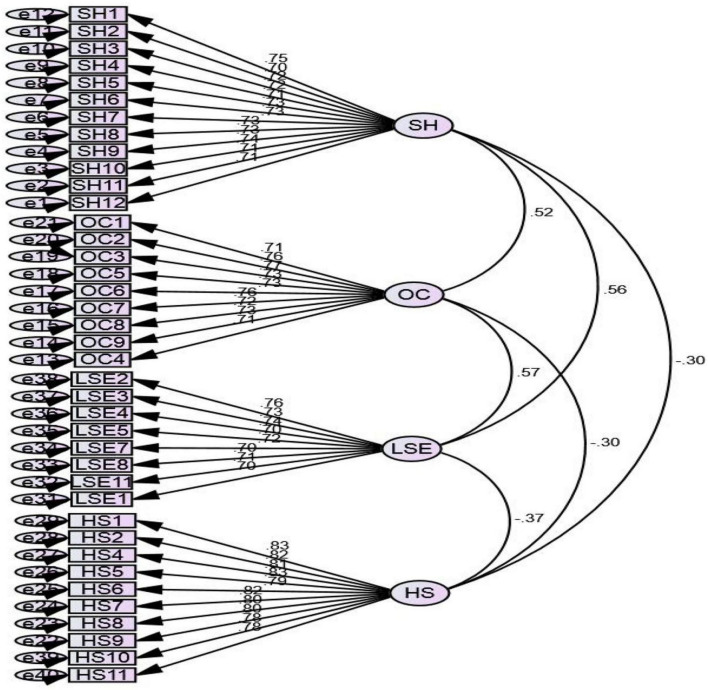
Factor loadings of four items.

Our study utilized the CFA for the SH model, as shown in Table 1A. Items with standardized factor loadings and covariance among the variables are plotted in Figure 1. The regression weights were significant (*p* < 0.01) and have standardized loading > 0.50. The model showed that acceptable evidence fulfills the criteria for model fit measure indexes, as the model goodness-of-fit indexes [global fit index (GFI) > 0.90] (Jöreskog and Sörbom, 1993) are measured using the maximum likelihood (oblique rotation) function for the confirmation of hypothesized model. The study found that CMIN/DF ratio value between 1 and 3, i.e., (χ^2^/df = 1.41), lies in the acceptance criteria, and chi-square has the significant value [χ^2^ (CMIN) = 979.17, *p* < 0.05] (Marsh and Hocevar, 1985) at 5% CI. The next index measured was the comparative fit index (CFI), which has a value of 0.97 (> 0.90). Root mean square error of approximation (RMSEA) found a value of 0.03, which was < 0.07 (threshold criteria) (Browne and Cudeck, 1993). Other baseline criteria for model fitness [SRMR = 0.05 < 0.08 (Browne and Cudeck, 1993); the incremental fit index (IFI) = 0.97 > 0.95; Tucker-Lewis index (TLI) = 0.96 > 0.95) also met the acceptance criteria (Bentler, 1990).

**TABLE 1A:** Standardized factor loadings, composite reliability and average variance extracted of four factors model.

			Estimate	Composite reliability	Average variance extracted
SH1	<—	SH	0.750	0.93	0.52
SH12	<—	SH	0.713		
SH10	<—	SH	0.739		
SH9	<—	SH	0.728		
SH8	<—	SH	0.731		
SH7	<—	SH	0.729		
SH6	<—	SH	0.732		
SH5	<—	SH	0.714		
SH2	<—	SH	0.701		
SH11	<—	SH	0.714		
SH4	<—	SH	0.721		
SH3	<—	SH	0.716		
					
LSE5	<—	LSE	0.705	0.90	0.51
LSE8	<—	LSE	0.696		
LSE11	<—	LSE	0.708		
LSE1	<—	LSE	0.703		
LSE7	<—	LSE	0.720		
LSE4	<—	LSE	0.740		
LSE3	<—	LSE	0.726		
LSE2	<—	LSE	0.759		
LSE5	<—	LSE	0.705		
LSE8	<—	LSE	0.696	0.94	0.64
HS1	<—	HS	0.832		
HS4	<—	HS	0.812		
HS6	<—	HS	0.787		
HS5	<—	HS	0.828		
HS2	<—	HS	0.820		
HS11	<—	HS	0.780		
HS8	<—	HS	0.795		
HS9	<—	HS	0.799		
HS10	<—	HS	0.776		
HS7	<—	HS	0.822		
OC5	<—	OC	0.735	0.91	0.54
OC8	<—	OC	0.724		
OC9	<—	OC	0.725		
OC7	<—	OC	0.764		
OC4	<—	OC	0.707		
OC6	<—	OC	0.732		
OC3	<—	OC	0.766		
OC2	<—	OC	0.763		
OC1	<—	OC	0.706		

**TABLE 1B:** Confirmatory factor analysis.

Measure	CMIN	DF	CMIN/DF	CFI	SRMR	RMSEA	P-Close	GFI	IFI	TLI
Estimate	979.17	696	1.41	0.97	0.05	0.03	1.00	0.89	0.97	0.96
Threshold	–	–	Between 1 and 3	>0.95	<0.08	<0.06	>0.05	>0.90	>0.95	>0.95

### Tests of reliability and validity

After eliminating the required items, the analysis was performed. The results are provided in Table 2. The internal consistency of items of each variable is measured by composite reliability using Cronbach’s alpha, which was > 0.7 threshold criteria (0.91 = OC, 0.90 = LSE, 0.94 = HS, and 0.93 = SH), and all AVE values were > 0.5 globally set standard (Chin, 2010), which means that they were acceptable according to the range of CR and AVE (see Table 2). By comparing the square root of the AVE of constructs with correlations of other constructs, we found that the off-diagonal correlation (ranging from −0.29 to 0.56) was less than the square root of AVE (ranging from 0.71 to 0.80), which confirms discriminant validity (Henseler et al., 2015).

**TABLE 2:** Descriptive statistics, correlation, and discriminant validity.

	CR	AVE	MSV	MaxR (H)	OC	SH	LSE	HS	TOL	VIF
OC	0.91	0.54	0.32	0.91	0.73				0.66	1.49
SH	0.93	0.52	0.31	0.93	0.51***	0.72				
LSE	0.89	0.51	0.32	0.89	0.56***	0.56***	0.72		0.67	1.47
HS	0.94	0.64	0.13	0.94	–0.29***	–0.29***	–0.36***	0.80	0.89	1.11

Significance level: ***p < 0.001.

### Structural equation modeling

#### Multivariate normality

A Cook’s distance analysis was performed to determine if any (multivariate) influential outliers existed. Cook’s distance can be utilized in several ways. For example, it can be utilized to designate significant data points that are predominantly momentous inspecting for validity or to point out areas of the design space where it would be appropriate to be able to obtain additional data points (Hossin et al., 2021).

#### Collinearity assessment

In general, variance inflation factors (VIFs) can have a range from 1 to 10. The VIF can explain the percentage of variance inflated for every coefficient. While interpreting the VIFs, usually 1 represents not correlated, 1–5 indicates moderately correlated, and 5–10 shows highly correlated (Hossin et al., 2021); there was no issue of multicollinearity as the variance inflation factor (VIF) of the model was less than the cut-off criteria of 5.0 and tolerance > 0.1 (Byrne, 2010; Bagozzi and Yi, 2012).

#### Standardized results

**FIGURE 2:**
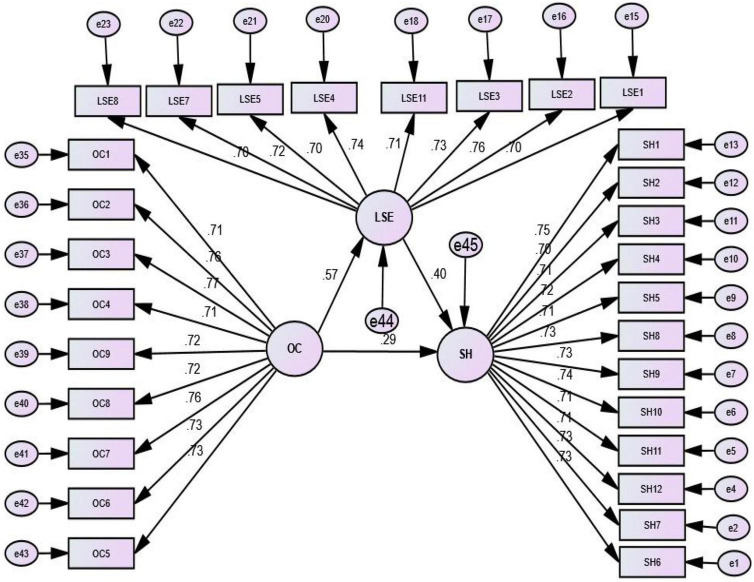
Relationship between organizational climate (OC) and sexual harassment (SH) by the mediator.

The standardized results of the path coefficients, through simple regression analysis, showed that OC has a positive and significant direct linear relationship with SH (β = 0.49, *CR* = 9.06, *p* < 0.001). While incorporating mediator LSE, the direct effect of OC on SH was positive and significant (β = 0.29, *CR* = 4.98, *p* < 0.01), whereas in the second path, OC has a significant relationship with LSE (β = 0.60, *CR* = 9.56, *p* < 0.001) and LSE has a significant relationship with SH (β = 0.37, *CR* = 6.57, *p* < 0.001) (see Table 3). These results are also represented in a diagram (see Figure 2).

**TABLE 3:** Standardized regression weights.

Outcome		Predictor	Estimate	S.E.	C.R.	*P*	Path
LSE	<—	OC	0.60	0.06	9.56	***	A
SH	<—	OC	0.29	0.05	4.98	***	B
SH	<—	LSE	0.37	0.05	6.57	***	
Total Effect	SH<–OC	0.51**					
Direct Effect	SH<–OC	0.29**					
Indirect Effect	SH<–OC	0.22***					

**Parameter**	**Estimate**		**Lower**	**Upper**			***P***

A x B	0.225		0.162	0.307			0.000

Significance level: **p < 0.010, ***p < 0.00.

### Indirect effects

The results of the total effect (sum of direct and indirect effects) of OC on SH using bootstrapping 5,000 re-sample showed positive and significant links (total effect = 0.51, *p* < 0.01). In the direct path, OC was positively related to SH (direct effect = 0.29, *p* < 0.01). The conditional indirect path OC to SH through mediator LSE was also positive and significant (indirect effect = 0.22, *p* < 0.001), which confirms hypothesis 2. Results presented in Table 3 reveal a partial mediation as the impact of LSE on the relationship between OC and SH is significant (Cheung and Lau, 2008).

### Moderation effects

**FIGURE 3:**
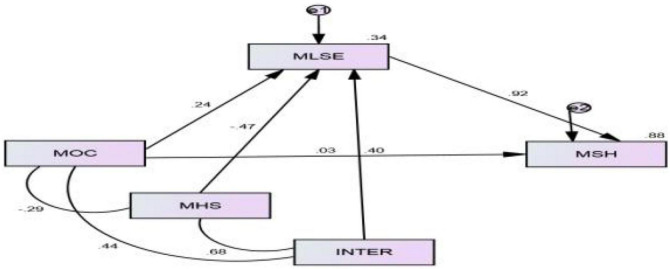
Structural equation modeling (SEM).

**FIGURE 4:**
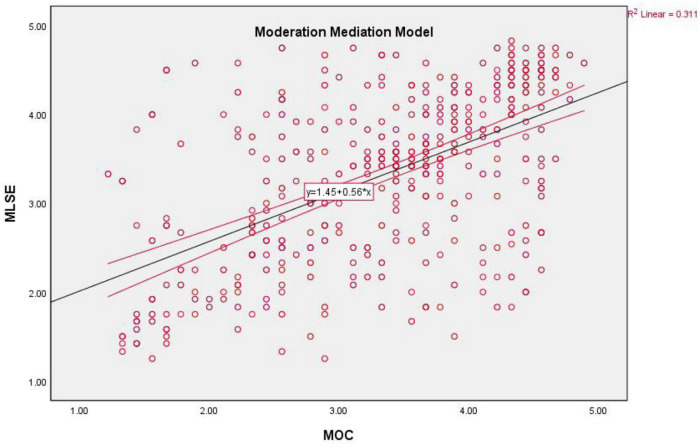
Graphical representation of moderated mediation model.

The results described that the interaction term has a positive and significant relationship with LSE (β = + 0.10, *CR* = 2.87, *p* = 0.01) (see Table 4). Results confirm (as we assumed) that HS moderates significantly as the interaction between OC and HS was significant. R-square showed that results could explain a 34% variation in SH through the moderated mediation effect and expected a 1% change in the R-square due to moderator (see Figure 3). The interaction was probed by testing the conditional effects of OS at three levels of HS: one standard deviation below the mean (1.66), one standard deviation at the mean (2.68), and one standard deviation above the mean (3.70). The conditional effect of the low and high values of the moderator increases as the values increase, and the positive relationship between perceived OS and LSE becomes stronger. When the value of HS decreases (−1 SD), there was no effect of organizational structure on LSE (Figure 4). Hence, all the hypotheses were true.

**TABLE 4:** Moderated mediation effect on sexual harassment.

			Estimate	S.E.	C.R.	*P*	*t*_value	Label
ZMLSE	<—	ZMOC	0.24	0.10	2.28	0.022	12.11	R- -Sqr = 34% Change in R-Sqr = 0.01***
ZMLSE	<—	ZMHS	–0.46	0.13	–3.59	***	–0.17	
ZMLSE	<—	OC_HS	0.10	0.037	2.87	0.004	2.86	
ZMSH	<—	ZMOC	0.030	0.020	1.49	0.135	1.49	
ZMSH	<—	ZMLSE	0.92	0.020	46.38	***	46.28	

**Index**			**Boot LLCI**				**Boot ULCI**	**Boot SE**

**Index of moderation mediation**					
0.09			0.04				0.16	0.02

Significance level: *p* < 0.001***.

## Discussion

After 50 decades in 2010, Pakistan’s parliament passed the Protection against Harassment for Women at Work Act 2010, while Pakistan has also ratified the United Nations laws against women’s aggression, rendering it illegal to make any sound, show an object, utter a word or demand sexual favors deemed SH. The other legislation, 509 Code of Criminal Procedure covering private meetings, tackles sexual assault in public places and encompasses all spaces outside the workplace, and it enables an advanced understanding regarding fundamental reasons for SH of athletes in sports settings, i.e., OC, HS, and LSE. The study aimed to understand why and how the perception of an athlete of OC can predict SH by extending an indirect effect of the underlying psychological mechanisms of LSE and the moderating role of HS because HS paves the way for someone who indulges in SH. Results found a positive and significant relationship between OC and SH. The results are in line with Cleveland et al. (2000) who stated that particular traits of OC, such as gendered interpersonal norms, may lead men to regard women exclusively as sex objects. In this way, women’s perceptions of SH in a given country can be related to their status there (Hausen, 2010), because of their lower strength and quickness (Gul et al., 2019). For example, coaches are thriving in SH incidence while athletes have lost their professions and their social prestige (Jahangir and Manzoor, 2018). In reality, patriarchs in Pakistani society considered that problems concerning women in sports are trivial and unimportant (Gul et al., 2019).

This study found that LSE mediated the relationship between OC and SH. The findings are in line with Burn’s (2019) study which stated that, whether purposely or inadvertently, the creation of an intimidating, hostile, and violent environment undermines the confidence of the targets of SH and makes it more difficult for the targets to achieve their goals. Kidnapping, nepotism, favoritism, brutality, and SH are only a few of the ills that have developed in our culture (Manzoor et al., 2019). Furthermore, fear of being accused of approving of the SH, fear of being accused of inventing the entire thing or of seeking personal benefits, about where to obtain support, and a preference of silence (Esapzai, 2014) all together create an atmosphere that feminists would consider perfect for SH, and also, athletes are showing less confidence compared to developed countries’ female athletes (Gul et al., 2019). For the sake of their position, few experienced sportswomen allow coaches, managers, and selectors to use young girls (Jahangir and Manzoor, 2018). The women dose not raise their voice against SH. Recently, Hamza Mukhtar filed SH charges against the cricketer Babar Azam, but he stated that he was used to it (Firstpost, 2021). Do the organizations define sexual harassment and outline unacceptable behavior and its consequences in an effective way? In this regard, this study found that HS moderated the relationship between OC and LSE. Wiener et al. (1997) conducted a study to examine the relationship between athletes’ ambivalent sexism and their perception of SH. They reported that female athletes who have low levels of HS perceived the harasser’s conduct as more severe than those who have high levels of HS. In line with Laar et al. (2019), the cultural component of hostility is of tremendous significance. Moreover, in a patriarchal society, if a female participant openly competes with male participants in any athletic activity, this is considered disgraceful and indecent, considering the cultural practices where women are regulated under religion in a patriarchal society (Ashraf, 2019). This study has advanced the knowledge in the field by adding a new variable of “hostile sexism” in the OC model. The researcher can use this finding in other law enforcement agencies and other traditional women’s organizations. So, the overall model predictive ability was obtained by moderated mediation model. HS significantly moderates the perception of female athletes of OC and LSE. Findings reveal that SH against females is fueled by men’s attempts to secure power and dominance. Jaffa Football Club in Pakistan is suffering because of female players, as the local political forces want women to get away from football, so they reportedly started bullying them sexually. Findings are in line with Begany and Milburn (2002) who stated that men’s likelihood of engaging in SH is due to their belief in rape and authoritarianism. Furthermore, Weiner et al. (1997) stated that males and females having a higher perception of HS appear to experience more behavior of SH as compared to benevolent sexism. Additionally, Sibley Butler and Schmidtke (2010) found that SH happens as a result of conventional sex-role expectations among both men and women. Society expects men to be aggressors. The normative presumption is that men are rewarded by honor for this attitude. Feminists believe that women’s voices are not exposed to the light in general society because the patriarchy negatively influences their lives (Kim et al., 2016). Moreover, significant mediation of LSE reveals that when an athlete reacts to SH, organizations take no action on the complaint but rather say that athletic workers should undergo annual instruction. Women are discouraged from participating in athletics due to social concerns, cultural barriers, poverty, and a lack of access to education. Also, SH is a barrier in the way of their success. We hypothesized that some factors influence this behavior, and consequently, all the hypotheses are accepted.

## Conclusion and recommendations

By showing the fact that SH is a complicated issue that is influenced by some factors, this study supports the predictions of the SH theories from a feminist viewpoint. The study aims to reveal the athletes’ perceptions of SH and OC impact on SH, as well as the mediating mechanisms of LSE and the moderating role of HS. Results indicate the seriousness of the situation, with a significant focus on the positions of relevant authorities, where OC is a contributing factor in the study area and is significantly associated with athletes’ perception of SH behavior. SH suggests that women are treated less favorably in the workplace (sports) and society as a whole than men, and as a result, LSE partially mediates the positive effects of athletes’ perceptions of OC on SH. It is a sign of imbalance of power and male dominance (including coaches and male athletes) that promotes multiple types of violence and SH against female athletes (Dworkin and Messner, 2002), where HS strengthens the perceived relationship between OC and LSE, which stimulates the SH of athletes. Therefore, methods of monitoring, documenting, and reporting SH are necessary to improve the environment (Leaper and Brown, 2008). Programs that encourage bystander intervention are also crucial for minimizing SH. In addition to the costly court system and entrenched power relations, vibrant policy ideas are therefore vital to eradicate SH. In coaching, men have traditionally been viewed as the norm, enabling men to alter the normative male environments that encourage SH (Gidycz et al., 2011). All organizations are mandated to provide committees of inquiry to deal with grievances, and the provincial and federal ombudsman’s offices can be contacted directly by women. The patriarchal structure is profoundly rooted in the fact that it will take decades to alter. The efficacy of law enforcement in socializing gender equality is also necessary. Once sports committees properly enforce all of the ramifications by looking at the past analyses of the occurrence of SH, a dramatic reduction in the amount of SH of women in sports could be seen.

## Limitations and future directions

This study presents both theoretical and practical implications as well as charts the areas of further research. SH knowledge about the situation in Pakistan can be rewarding and a significant contribution to the literature. However, there are multiple drawbacks to this research. The information was gathered using the existing scale of ambivalence sexism, which has already been used in Turkey (Salman and Turgut, 2007), and no prior study has used it in Pakistan simply adding the fact of the culture which inevitably brings the danger of general bias. While female-to-female and male-to-male SH do exist, this study only investigates male-to-female SH in Pakistan’s women’s football sports clubs, with all three dimensions of SH in one construct. This study investigated causal relationships between variables based on a causal-phase approach. Cross-cultural analyses are, therefore, necessary to discern the causal course. This study investigated how OC interacts with an individual characteristic of self-esteem that fuels cultural factors of HS. Further research is required regarding the effects of these variables on the well-being of athletes and the impact of policy on identifying and reducing SH.

In Pakistan, moral and ethical dilemmas are closely linked to the ideas of women’s faith and virtue. Thus, measuring and under-reporting have been a crucial challenge in this problem, and likewise accessing organizations and athletes. Power relations also influence cooperation from organizations in data collection. In particular, this was related to a research carried out on SH. SH was not mentioned in the questionnaire, which allowed measuring the participants while maintaining ethical standards subjectively. An analysis that would involve interview-based assessment of elite athletes is also recommended.

## Data availability statement

The raw data supporting the conclusions of this article will be made available by the authors, without undue reservation.

## Ethics statement

The studies involving human participants were reviewed and approved by South China Normal University and School of Physical Education and Sports Sciences. The patients/participants provided their written informed consent to participate in this study.

## Author contributions

The author confirms being the sole contributor of this work and has approved it for publication.
