# Supplementary material for: Factors influencing sexual harassment behavior in sports environment: Evidence from Pakistan
Source: Front Psychol. 2022 Nov 17;13:837078. doi: 10.3389/fpsyg.2022.837078 (PMC9713023; doi:10.3389/fpsyg.2022.837078)
Supplement: Supplementary file 1 [file Table_1.docx]

**Supplementary Table 1; QUESTIONNAIRE**

| ***LOW SELF ESTEEM*** |
| --- |
| Do you think some players feel comfortable when approaching someone in a position to get better grades or promotion. |
| Females less confidently approach and deal with perpetrator behaviour. |
| Do you think Females feel insecure in social situation often blame themselves. |
| I frequently find it difficult to defend myself and confronted coaches |
| I usually do what the others want i am less confident to correctly classify man behavior. |
| I feel my appearance makes a man to increase perceptions of unwanted sexual attention |
| looking other people in the eye and reveal the sexual harasser is difficult |
| I am afraid of others being labelled me bad women. |
| Females are forced to engage in a sexual act with the promise of a reward? |
| In a group of people I feel trouble to say the right things. |
| Females less confidently approach and deal with perpetrator behaviour. |
| Females enjoy social gatherings just to be with people. |
| Females would be willing to describe myself as a pretty "strong" personality. |
| ***HOSTILE SEXISM*** |
| Feminists are not seeking for women to have more power than men. |
| Many women are actually seeking special favors, such as hiring policies that  Favour them over men, under the guise of asking for “equality. |
| Most women interpret innocent remarks or acts as being sexist. |
| Women are too easily offended. |
| Most women fail to appreciate fully all that men do for them. |
| Women seek to gain power by getting control over men. |
| Women exaggerate problems they have at work. |
| Once a woman gets a man to commit to her, she usually tries to put him on |
| When women lose to men in a fair competition, they typically complain |
| There are actually very few women who get a kick out of teasing men by |
| Feminists are making entirely reasonable demands of men. |
| ***ORGANISATIONAL CLIMATE*** |
| My organization doesn’t want employees to come forward about sexual harassment |
| My organization discourages employees from talking about instances of sexual harassment at |
| My organization takes sexual harassment seriously. |
| There could be professional consequences for the victim if they report sexual harassment. |
| My organization is willing to take action against employees who sexually harass others. |
| Do you agree that The leadership at sports organization enforces its policy against sexual |
| I felt silenced at my organization. |
| It is risky to report sexual harassment in my organization. |
| The process of reporting a formal complaint of sexual harassment at my workplace is |
| ***SEXUAL HARASSMENT*** |
| female players encounter offensive sexual remarks about their dress, body, or behavior. |
| female players encounter offensive and sexist remarks and body gestures. |
| female players are leered, or ogled in a way that makes them uncomfortable. |
| female players are repeatedly told offensive sexual stories or jokes. |
| Sexist or suggestive materials are displayed to women players. |
| female players repeatedly recieved emails, tweets, phone, or instant messages, pictures or videos, that made them feel insecure? |
| female players are touched in a way that makes them uncomfortable. |
| female players are fondle without consent? |
| female players encounter unwanted attempts to establish a romantic sexual relationship. |
| Attempts are made to have talk on sexual matters with female players without their consent. |
| female players encounter threats to be treated badly if did not cooperate sexually. |
| Offered to be sexually cooperative to you in exchanged for a favor or special treatment from you . |
| Touched you in a way that made you feel uncomfortable |
